# Recent Advances in the Role of Nuclear Factor Erythroid-2-Related Factor 2 in Spinal Cord Injury: Regulatory Mechanisms and Therapeutic Options

**DOI:** 10.3389/fnagi.2022.851257

**Published:** 2022-06-10

**Authors:** Tianqi Jiang, Yongxiong He

**Affiliations:** ^1^Graduate School of Inner Mongolia Medical University, Hohhot, China; ^2^Spine Surgery, Inner Mongolia People’s Hospital, Hohhot, China

**Keywords:** Nrf2, SCI, oxidative stress, inflammatory injury, treatment

## Abstract

Nuclear factor erythroid-2-related factor 2 (Nrf2) is a pleiotropic transcription factor, and it has been documented that it can induce defense mechanisms both oxidative stress and inflammatory injury. At present, more and more evidences show that the Nrf2 signaling pathway is a key pharmacological target for the treatment of spinal cord injury (SCI), and activating the Nrf2 signaling pathway can effectively treat the inflammatory injury and oxidative stress after SCI. This article firstly introduces the biological studies of the Nrf2 pathway. Meanwhile, it is more powerful to explain that activating the Nrf2 signaling pathway can effectively treat SCI by deeply exploring the relationship between Nrf2 and oxidative stress, inflammatory injury, and SCI. In addition, several potential drugs for the treatment of SCI by promoting Nrf2 activation and Nrf2-dependent gene expression are reviewed. And some other treatment strategies of SCI by modulating the Nrf2 pathway are also summarized. It will provide new ideas and directions for the treatment of SCI.

## Introduction

Spinal cord injury (SCI) is a serious complication of spinal injury, which causes loss of motor, sensory and physiological functions below the injured segment. It is often due to traumatic or non-traumatic reasons, causing spinal fracture or dislocation and then SCI (Karsy and Hawryluk, 2019; Liu et al., 2021a). The emergence of SCI brings long-term physical, psychological and economic pressure to patients and their families (Chay and Kirshblum, 2020). The global incidence of SCI is estimated to reach 40–80 per million people, and it is increasing year by year with the increase in car or extreme sports accidents (Zawadzka et al., 2021). However, there are significant regional differences in the incidence and prevalence of SCI in that this disease has only been systematically studied in developed countries, there are inadequate studies on SCI in most developing countries. Therefore, it is difficult to accurately count the number and distribution of patients with SCI in the world (Hamid et al., 2018).

For SCI, the initial trauma directly leads to the destruction of the spinal structure, which in turn causes the spinal cord to be compressed or injured. Necrosis of neurons and oligodendrocytes and destruction of the vasculature and blood-spinal cord barrier (BSCB) appear in the injured spinal cord. These events will immediately trigger a series of secondary injuries, such as inflammatory injury, cell edema, cell apoptosis, oxidative stress, tissue ischemia, etc., leading to further damage to the spinal cord and nerves and causing physiological dysfunction (Al Mamun et al., 2021). Neurons, as a kind of non-renewable cells, are easily affected by peripheral cytokines. After the spinal cord being injured, the function of the central nervous system cannot be restored. This is the main reason why SCI has become a clinically incurable disease (Zhang C. et al., 2021). As we all know, all the efforts and attempts made to effectively treat SCI are to find an efficient and long-term treatment method, but unfortunately, there is currently no exact treatment strategy that can achieve this goal (Zhang Y. et al., 2021). Primary injury triggers secondary injury, which produces further chemical and mechanical damage to spinal cord tissue, and involves pathological changes at the cellular and molecular levels, and this process is reversible (Liu et al., 2021a). Therefore, the most ideal approach in the treatment of spinal cord injury is to inhibit secondary injury and promote functional recovery. In the secondary injury, due to the role of inflammation and oxidative stress in the pathogenesis of SCI, inhibition of oxidative stress and inflammation may be a suitable strategy for alleviating SCI (Samarghandian et al., 2020). Nowadays, many eyes are now focused on the signal pathway, hoping to find a more effective way to treat SCI. A large amount of experimental studies have found that a transcription factor, nuclear factor erythroid-2-related factor 2 (Nrf2), is involved in the pathogenesis of SCI and easily responds to traumatic injuries. Therefore, increasing the expression of Nrf2 through gene therapy may be a feasible SCI repair strategy (Kanninen et al., 2015). This article deeply analyzes the mechanism of Nrf2 as the main regulator of anti-oxidative stress and inflammatory damage, and summarizes the medication and other treatment modalities based on the regulation of the Nrf2 pathway, with the hope that it will be beneficial to the development of more effective treatments for SCI in the future.

## The Occurrence and Development of Spinal Cord Injury

Spinal cord injury is divided into two stages, primary and secondary. Primary injury is irreversible, including spinal cord concussion, spinal cord contusion, vascular disorders, cell death, spinal cord axon growth inhibition, etc. (Liu et al., 2021a). Primary injury will immediately trigger a continuous secondary injury cascade reaction, including increased cell permeability, edema, apoptotic signaling, ischemia, excitotoxicity, vascular injury, inflammatory injury, demyelination, etc., which eventually will lead to neuronal damage and death (Anjum et al., 2020). However, the complex pathophysiological changes of SCI make the secondary injury more lethal than the primary injury. Therefore, the primary condition for the treatment of SCI is to control the secondary injury.

## Biological Study of Nuclear Factor Erythroid-2-Related Factor 2 Pathway

### The Structure of Nuclear Factor Erythroid-2-Related Factor 2

**FIGURE 1:**
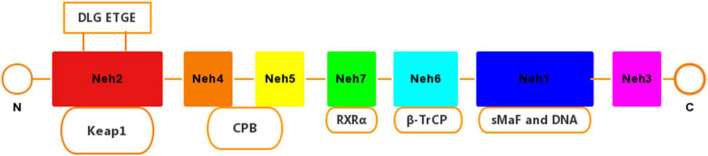
Domain structures of Nrf2. The Nrf2 protein contains seven domains, Nehl–Neh7. The ETGE and DLG motifs in the Neh2 domain are essential for the direct interaction with the Kelch domain of Keap1.

Nuclear factor erythroid-2-related factor 2 is a pleiotropic transcription factor containing 605 amino acid residues, which can induce a defense mechanism against oxidative stress and inflammatory damage, and regulate the expression of related genes. Nrf2 is composed of 7 functional domains (Neh1–Neh7) (Figure 1). Among them, the Neh1 domain has a cap‘n’collar basic-region leucine zipper (BZIP) domain, which regulates DNA-binding (Sun et al., 2009) and a nuclear localization signal (NLS) that is responsible for the nuclear translocation of Nrf2 (Theodore et al., 2008). The Neh2 domain has two binding sites with Kelch-like ECH-associated protein 1 (Keap1), which combine to form a homodimer (Stefanson and Bakovic, 2014). Neh3 acts as a transactivation domain to interact with CHD6 (a chromo-ATPase/helicase DNA binding protein) (Nioi et al., 2005). Neh4 and Neh5 combine to assist the activator cyclic adenosine monophosphate (AMP) response element binding protein to promote Nrf2 transcription. In addition, Neh4 and Neh5 can also interact with the nuclear cofactor RAC3/AIB1/SRC-3, thereby enhancing the expression of antioxidant response element (ARE) genes targeting Nrf2 (Kil et al., 1999; Schwartz and Yoles, 2006). The Neh7 domain can repress Nrf2 by linking up with retinoic X receptor alpha (RXRα) (Tu et al., 2019).

### Keap1-Dependent Regulation of Nuclear Factor Erythroid-2-Related Factor 2 Activity

In the inactivated state, Nrf2 is interdependent in the cytoplasm through Neh2 and Keap1. Keap1 binds to Nrf2 in the cytoplasm to promote the ubiquitination and degradation of Nrf2 (Suzuki and Yamamoto, 2015). Keap1 has 5 active cysteine residues (Cys), each of which can induce chemical reactions through cysteine thiol, so that Nrf2 is released from Keap1 (Baird and Dinkova-Kostova, 2011). When stimulated by oxidative stress or electrophilic substances, a cysteine in Keap1 is oxidized and releases Nrf2 into the nucleus. The released Nrf2 undergoes nuclear translocation and binds to the ARE (Itoh et al., 1997; Keum and Choi, 2014), thereby initiating the transcription of downstream genes (Itoh et al., 1997; Vargas et al., 2008; Pall and Levine, 2015). At present, although several mechanisms have been proposed, the molecular mechanism describing how Nrf2 escapes the control of Keap1 is not fully understood. One of the mechanisms involves the modification of cysteine within Keap1 (Baird and Dinkova-Kostova, 2011). The different chemical substances that trigger the Keap1-Nrf2 system are related to the different modes of Keap1 cysteine modification, and the cysteine code can respond to a variety of chemical substances and oxidative damage (Suzuki and Yamamoto, 2015). The key cysteine residues that may play a role in Keap1 have been identified. Cys151 cysteine residues have been shown to be sensors (Zhang and Hannink, 2003) and are covalently modified by electrophilic species or reactive oxygen species (ROS) to interfere with Keap1 and Cul3. The interaction caused Nrf2 to separate from Keap1 (Zhang et al., 2004; Gao et al., 2007; Rachakonda et al., 2008). Studies by Kobayashi et al. (2006) have shown that the nuclear accumulation of Nrf2 requires *de novo* protein synthesis. Cys273 and Cys288 residues that maintain Keap1 activity participate in the ubiquitin-proteasome degradation mechanism of Nrf2, but do not regulate the binding or dissociation of Nrf2 and Keap1. The modification of these cysteine residues will inhibit the ubiquitin coupling of the Keap1-cul3 complex with Nrf2, stimulate the opening of the Keap1 gate, and lead to the accumulation of Nrf2 in the nucleus (Kobayashi et al., 2006). A more widely convincing model proposes a Keap1 hinge and latch dissociation mechanism. That is Nrf2 binds to the Keap1 homodimer through a high-affinity ETGE motif as the “hinge” and a low-affinity DLG motif as the “latch.” This causes the conformational change of Keap1, which is the basis of the Nrf2 activation “latch and hinge” theory (Tong et al., 2007; Hast et al., 2013). The “hinge and latch” model was originally based on *in vitro* biochemical and structural evidence, which came from studies using purified peptides (Eggler et al., 2005; Tong et al., 2006; Shibata et al., 2008). This model is supported by cancer-related somatic mutations that specifically change the amino acids in the DLG or ETGE motif, leading to abnormal cell accumulation of Nrf2 (Shibata et al., 2008; Sykiotis and Bohmann, 2010). When the cell returns to a stable state, karyopherin alpha 6 (importin alpha 7) (KPNA6) translocates Keap1 to the nucleus, thereby “turning off” the transcription of Nrf2 and restoring the Keap1 mediated ubiquitination and degradation mechanism (Audousset et al., 2021). At the same time, the chemometric measurement results support the ratio of Keap1 and Nrf2 in the complex to 2:1 (Lo et al., 2006). In addition, studies have shown that p21 protein directly interacts with DLG and ETGE motifs, thereby competing with Keap1 for binding to Nrf2 (Chen et al., 2009).

### Keap1-Independently Regulates Nuclear Factor Erythroid-2-Related Factor 2 Activity

Three E3 ubiquitin ligase complexes, (1) βTrCP-S-phase kinase-associated protein-1 (Skp1)-Cul1-Rbx1, (2) HMG-CoA reductase degradation 1 (Hrd1) and (3) WD-repeat protein 23(WDR23)-Cul4-damaged DNA binding protein 1 (DDB1), are known to be involved in Keap1-independent Nrf2 degradation. DSGIS and DSAPGS degron of the Neh6 domain combine to form Trp-Asp (W-D) dipeptide repeat sequence (WD40), and form a ubiquitin ligase complex with Skp1, Cul1, and Rbx1 proteins (Zgorzynska et al., 2021). Research by Rada et al. (2011) found that the phosphorylation event of Neh6 produces a phosphorylation destruction motif, and the β-trcp-skp1-cul1-rbx1 E3 ubiquitin ligase complex can then recognize the site. Protein kinases have also been shown to play an important role in Keap1-independent Nrf2 activation. Phosphorylation of specific amino acid residues in Nrf2 can increase its stability and transactivation activity (Nguyen et al., 2003). In addition, the activity of Nrf2 is regulated by the kinase that directly phosphorylates Nrf2. The protein kinase pathways that have been determined to be associated with Keap1’s independent activation of Nrf2 include phosphatidylinositol 3-kinase (PI3K), mitogen-activated protein kinases (MAPKs), protein kinase C (PKC) and glycogen synthase kinase 3 (GSK-3) (Liu et al., 2019). Among them, GSK-3 phosphorylation of the DSGIS motif in Nrf2 can promote β-trcp to bind to Nrf2 (Chowdhry et al., 2013) and degrade Nrf2. The reason may be that phosphatase and tensin homolog (PTEN) work together to enhance this process (Rojo et al., 2014). Previous studies have found that GSK3 plays a key role in the regulation of Nrf2 by growth factors. GSK-3 promotes the degradation of Nrf2 through phosphorylation of DSGIS motif and SCFβ-TrCP to achieve the purpose of negative control of Nrf2. Promoting the activation of PKB/Akt, p90RSK, and PKC can inhibit GSK-3, thereby inhibiting the phosphorylation of DSGIS motifs and the degradation of Nrf2 (Hayes et al., 2015).

### Transcriptional Regulation of Nuclear Factor Erythroid-2-Related Factor 2

Studies have found that endogenous oncogenes such as K-RasG12D, B-RafV619E, and MycERT2 increase the transcription of Nrf2 (Denicola et al., 2011). Among them, the oncogenic activation and amplification process of the oncogene KRAS can activate the Nrf2-mediated protection mechanism, leading to chemotherapy resistance (Tao et al., 2014). In addition, experiments have shown that Nrf2 mRNA transcription is negatively regulated by microRNAs (miRNAs) (Zhao et al., 2018; Zhu et al., 2019; Jadeja et al., 2020). These non-coding RNAs regulate gene expression in a specific sequence, by enhancing target mRNA degradation or inhibiting translation (Breving and Esquela-Kerscher, 2010). Among them, the first miRNA found to negatively regulate Nrf2 is miR-144. Studies have shown that miR-144 can reduce the content of Nrf2 in pathological red blood cells (Sangokoya et al., 2010). At the same time, miRNA may also promote the activation of Nrf2 to promote cell survival under stress conditions (Zgorzynska et al., 2021). Experiments have pointed out that miRNA-380-5p promotes the activation of the Nrf2-Keap1 signaling pathway by directly inhibiting Bach1 (Wang and Xu, 2021). In addition, the overexpression of miR-152-3p directly inhibits postsynaptic density protein 93 (PSD-93) so as to enhance the Nrf2/ARE signaling pathway, and reduces the damage of oxygen glucose deprivation/reoxygenation (OGD/R) cells (Zhang A. et al., 2019).

## The Relationship Between Nuclear Factor Erythroid-2-Related Factor 2 and Inflammation

Inflammatory damage is a protective mechanism produced by the body in response to external infections and tissue damage. Studies have shown that Nrf2 can protect cells and tissues damaged by inflammatory damage by regulating pro-inflammatory factors (Chen et al., 2006; Arisawa et al., 2007). Ma et al. (2006) tested mice with targeted interruption of Nrf2 gene and found that mice lacking Nrf2 developed lupus-like autoimmune syndrome, intravascular immunoglobulin complex deposition, and rapidly developing membrane proliferative glomerulonephritis, indicating that Nrf2 plays an important role in the process of inflammatory injury. In addition, Nrf2 itself can negatively regulate inflammatory mediators, such as chemokines, cytokines, cyclooxygenase 2 (COX-2), iNOS, etc., and reduce the activity of nuclear factor kappa-B (NF-κB) to achieve anti-inflammatory effects (Kim et al., 2010; Pedruzzi et al., 2012; Buelna-Chontal and Zazueta, 2013). Research by Wang et al. (2012) showed that sulforaphane (SF) was used to treat injured rats. During this process, the levels of Nrf2 and glutamate-cysteine ligase (GCL) increased significantly, and the level of inflammation have declined at the same time (Wang et al., 2012). In addition, inflammatory damage usually occurs within a few hours after the occurrence of SCI, and will cause the death of a large number of neurons. Studies have shown that SF reduces the activity of NF-κB and increases the activity of Nrf2, leading to the up-regulation of antioxidant enzymes and detoxification enzymes, thereby reducing the inflammatory damage of damaged spinal cord tissue and improving the state of nerve function (Mao et al., 2011).

## The Relationship Between Nuclear Factor Erythroid-2-Related Factor 2 and Oxidative Stress

**FIGURE 2:**
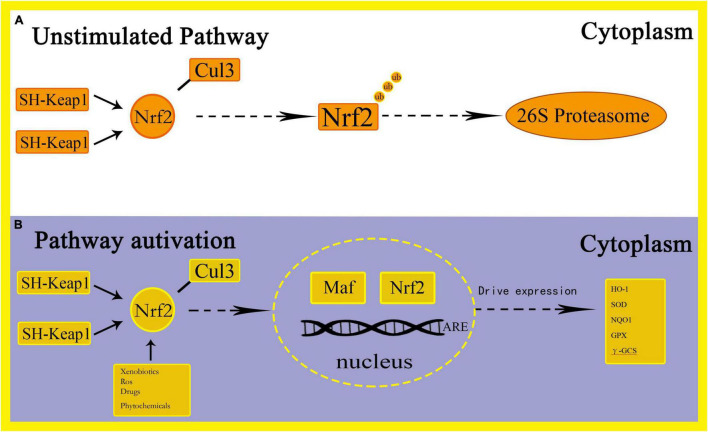
Schematic representation of Nrf2-ARE activation pathways.

Oxidative stress is defined as the imbalance between the production of free radicals and ROS. This imbalance can cause molecular and cell damage in the organism, and have a potential impact on the entire organism (Tu et al., 2019). The Nrf2 pathway is considered to be an important cellular defense mechanism against oxidative stress (Lee and Johnson, 2004; Kaspar et al., 2009; Catanzaro et al., 2017; Zhao et al., 2021). Studies have found that Nrf2 can induce expression of many cytoprotective genes during oxidative and electrophilic stress by regulating genes related to anti-oxidation mechanisms, such as the synthesis of glutathione (GSH), elimination of ROS, drug delivery, and detoxification of exogenous drugs (Taguchi et al., 2011). Among them, the increase in ROS levels can cause oxidative stress and activate pro-inflammatory pathways (Dreger et al., 2010). After oxidative stress, Nrf2 breaks away from Keap1 and enters the nucleus, combines with small Maf proteins to form a heterodimer, thereby targeting enhancer sequences regulated by ARE and driving the expression of antioxidant and drug metabolism enzyme genes, including HO-1, Superoxide dismutase (SOD), NAD(P)H:quinone oxidoreductase 1(NQO1), glutathione peroxidase (GPX) and γ-glutamylcysteine synthetase (γ-GCS), etc. (Hu et al., 2016; Krajka-Kuzniak et al., 2017) (Figure 2). Through this process, the purpose of increasing the activity of antioxidant enzymes, reducing the level of ROS and preventing oxidative damage is achieved (Mirzaei et al., 2021a). Among these genes, Nrf2 regulates the encoded heme oxygenase 1 (HO-1) to combat oxidative stress by removing the strong oxidant heme and increasing the levels of the endogenous antioxidants carbon monoxide and bilirubin (Prestera et al., 1995). In addition, NQO1 catalyzes the formation of hydroquinone from quinones through a single-step two-electron reduction reaction, thereby preventing the conversion of hydroquinone to ROS and achieving the function of preventing oxidative damage to DNA by environmental stressors (Jung and Kwak, 2010). The activity of Nrf2 is also regulated by the ubiquitin-binding protein P62. Under normal circumstances, P62 is degraded by autophagy. Under the action of oxidative stress, the content of P62 is up-regulated, thereby activating the expression of Nrf2 and Nrf2-dependent antioxidant defense genes (Bellezza et al., 2018). In addition, the occurrence of oxidative stress will also activate NFκB, leading to the up-regulation of P62 and the formation of TNF receptor-related factor 6 (TRAF6) complexes, thereby turning on the expression of antioxidant defense genes (White, 2012). Stimulating the Nrf2 antioxidant pathway to protect neurons has also been reported in other experimental environments, such as neuroinflammation (Linker et al., 2011). Studies have found that under oxidative stress conditions, Nrf2 can protect the outer mitochondrial membrane from oxidative damage (Strom et al., 2016). In addition, Mirzaei et al. (2021b) demonstrated that by inducing Nrf2 and exerting the anti-oxidative stress ability of Nrf2, it protects cells from cell death and alleviates the side effects of Doxorubicin in tumor patients. The study found that resveratrol can improve the phosphorylation level of p38, increase the content of Nrf2 in the nucleus and promote the expression of HO-1, which causes a significant reduction in oxidative stress and apoptosis, thereby improving cognition and spatial memory in mice (Farkhondeh et al., 2020).

## The Relationship Between Nuclear Factor Erythroid-2-Related Factor 2 and Spinal Cord Injury

The Nrf2 activates more than 24 genes to increase the antioxidant activity of tissue cells, so as to achieve the purpose of anti-inflammatory. At the same time, it can improve mitochondrial function and stimulate autophagy. Against oxidative stress, inflammatory damage and improving mitochondrial function, these three effects of Nrf2 may be applied to the treatment of dozens of chronic inflammatory diseases (Arisawa et al., 2007). Nrf2 responds to environmental stressors by activating a large number of antioxidant enzymes, detoxification enzymes and cytoprotective enzymes, and by restoring cell homeostasis. At the same time, Nrf2 also contributes to constitutive gene expression, which has been proven in cell transfection and *in vivo* studies on Nrf2−/− mice (Itoh et al., 1997; McMahon et al., 2001; Paladino et al., 2018). As Nrf2 is the master regulator of many protective genes, it may be a therapeutic target for some neurological diseases, including Alzheimer’s disease, SCI, Parkinson’s disease, Huntington’s disease, amyotrophic lateral sclerosis, and stroke, etc. (Samarghandian et al., 2020).

Some animal experiments have previously found that the Nrf2 signaling pathway plays an important role in spinal cord injury. Mao et al. (2012) compared the different effects of Nrf2 on wild-type and Nrf2 knockout mice after SCI. The experimental results reported that Nrf2 knockout mice developed severer hindlimb motor dysfunction and neuronal death after spinal cord injury. The elevated levels of IL-6 and IL-1 are considered to be the main reasons for the above experimental results. A later experiment by Mao et al. (2011) showed that SF activates Nrf2 in damaged spinal cord tissue, improves hindlimb motor function, and attenuates inflammatory damage and spinal cord edema in SCI mice. Wang et al. (2012) have experimentally confirmed that activation of the Nrf2/ARE pathway has neuroprotective effects on SCI. In experiments to determine the role of Nrf2 in the prevention of ozone excess-induced SCI, Zhang C. et al. (2021) found that tBHQ can activate the P62/Nrf2/antioxidant response element pathway in order to increase Nrf2 concentration, thereby enhancing the antioxidant system and protecting spinal cord neurons from high concentrations of ozone. In a clinically relevant model of spinal cord injury, Pomeshchik et al. (2014) found impaired hindlimb function in Nrf2−/− mice, manifested by spinal cord atrophy, demyelination, and increased astrogliosis, along with altered expression of genes that control apoptosis. After using lentiviral gene transfer, the expression of Nrf2 began to increase, and the function after spinal cord injury gradually recovered.

## The Therapeutic Application of Nuclear Factor Erythroid-2-Related Factor 2 in Spinal Cord Injury

### Nuclear Factor Erythroid-2-Related Factor 2 and Chinese Herbal Medicine

According to previous studies, many Chinese herbal medicines play an important role in the recovery of tissue cell function after SCI. Combined with conventional treatment and Chinese herbal medicine treatment, SCI can be treated more effectively. The following will focus on some of the Chinese herbal medicines that have been discovered in recent years by regulating Nrf2 to prevent oxidative stress and inflammatory damage to treat SCI. Table 1 shows the application of Nrf2 in Chinese Herbal Medicine.

**TABLE 1:** The therapeutic effects of Chinese herbal medicine on spinal cord injury.

Medicine	Model	Result	Mechanism	References
Polydatin	Rats	Keap1↓ Nrf2, NQO-1, and HO-1↑	Activate Nrf2/ARE pathway to reduce mitochondrial dysfunction	Zhan et al., 2021
Emodin	Rats	The expression of Nrf2, HO-1, GFAP and NF-κB protein in the emodin (20, 40, and 80 mg/kg) treatment group was higher than that of the control group	Activate Nrf2/ARE pathway	Zeng et al., 2018
Allicin	Rats	Recovery of motor function and neuronal damage in SCI rats	Nrf2 nuclear translocation in neurons and microglia	Runxiao et al., 2015
Salvianolic acid A	Rats	miR-101, Cul3, Nrf2, and HO-1?	Activate miR-101/Cul3/Nrf2/HO-1 signaling pathway	Yu et al., 2017
Salidroside	Rats	SOD activity↓, MDA, Nrf-2, and HO-1↑	Activate Nrf-2/HO-1 signaling pathway	Liu et al., 2021
Asiatic acid	Rats	IL-1β, IL-18, IL-6, TNF-α, ROS, H_2_O_2_, Malondialdehyde and NLRP3↓, SOD activity, GSH, Nrf2, and HO-1↑	Activate Nrf2 and HO-1 and inhibit ROS and NLRP3 pathways	Jiang et al., 2016
Curcumin	Rats	Nrf2 activity↑, NF-κBactivity↓	Activate Nrf2/HO-1 signaling pathway and inhibit P65	Jin W. et al., 2021
Sulforaphane	Rats	Nrf2 and HO-1↑	Increase the expression of Nrf2 and HO-1	Benedict et al., 2012
Rosmarinic acid	Rats	Nrf2, HO-1↑, TLR4, MyD88↓, IκB phosphorylation↓, NF-κB-p65 nuclear translocation↓	Regulates Nrf2/HO-1 and TLR4/NF-κ b pathways	Ma et al., 2020
Luteolin	Rats	Nrf2↑, NLRP3↓	Activate Nrf2 and inhibit NLRP3 pathway	Fu et al., 2018
Sinomenine	Rats	Nrf2 nuclear translocation↑, Nrf2-mediated transactivation↑	Activate Nrf2 signaling pathway	Zhang L. et al., 2019
Ginsenoside Rb1	Rats	Serum MDA↓, SOD, CAT, GSH activity↑, eNOS, HSP90, Nrf2, Nqo1, and HO-1↑	Activate eNOS/Nrf2/HO-1 pathway	Liu et al., 2018
Gastrodin	Rats	Expression of Nrf2, GCLc, and GCLm↑	Enhance Nrf2-GCLc/GCLm signal pathway	Du et al., 2016
Perillaldehyde	Rats	Nrf2and HO-1↑	Activate Nrf-2/HO-1 signaling pathway	Zheng W. et al., 2021
Trehalose	Rats	Nrf2 and HO-1↑	Activate the Nrf2/HO-1 signaling pathway	Gong et al., 2022

#### Polydatin

Polydatin (PD) is one of the important biologically active compounds in *Polygonum cuspidatum* and other medicinal plants. It has anti-cardiovascular, anti-inflammatory and anti-oxidant properties. Lv et al. (2019) studied the mechanism of PD alleviating SCI in rats. Studies have shown that PD may inhibit oxidative stress and apoptosis by regulating the Nrf2/HO-1 signal pathway of microglia, thereby protecting the spinal cord from SCI. Zhan et al. (2021) exposed spinal cord motor neurons (SMNs) to OGD/R environment to establish a spinal cord ischemia/reperfusion injury (SCII) model, which was treated with different doses of PD at different times. Experiments have found that PD can improve neuronal activity, and inhibit cell apoptosis and mitochondrial damage. It is concluded that PD protects mitochondrial function through the Nrf2/ARE signaling pathway and reduces OGD/R-induced neuronal damage and SCII.

#### Emodin

Emodin is an anthraquinone compound. It is widely used as a spice and traditional herb which exists in the rhizomes and roots of Polygonaceae plants *Rheum palmatum* and *Rheum tanguticum* in free and glycoside form. Emodin has been proven to have a variety of pharmacological effects, including anti-inflammatory, immunomodulatory, anti-fibrosis, anti-tumor, anti-viral, anti-bacterial, and anti-diabetic effects (Zheng Q. et al., 2021). Zeng et al. (2018) studied the effect of emodin on oxidative stress and inflammatory damage in acute SCI rats. The study found that the protein expression of Nrf2, HO-1, GFAP, and NF-κB after treatment with emodin (20, 40, and 80 mg/kg) was significantly higher than that of the control group. It is inferred that emodin has a protective effect on nerve cells after acute spinal cord injury. The mechanism is related to activating the Nrf2/ARE pathway, reducing the expression of NF-κB, ectodermal dysplasia-1(ED-1), tumor necrosis factor α (TNF-α), Interleukin-1β (IL-1β), Interleukin-6 (IL-6), and promoting the expression of GFAP and NG2.

#### Allicin

Allicin is an organic sulfur compound extracted from garlic heads. A large number of studies have found that allicin has strong antibacterial, anti-inflammatory, anti-fungal, lower plasma total cholesterol, affects insulin secretion, and detoxification, health care, anti-oxidation, anti-tumor and other pharmacology function (Bayan et al., 2014; Bhatwalkar et al., 2021). The results of Runxiao et al. (2015) showed that allicin promotes the recovery of motor function in SCI rats and protects neuronal damage. This effect may be related to its anti-oxidation, anti-inflammatory and anti-apoptotic effects. In addition, it was found that Nrf2 nuclear translocation in neurons and microglia after allicin treatment, it is speculated that the protective effect of allicin may also be related to Nrf2.

#### Salvianolic Acid A

Salvianolic acid A (Sal A) is a biologically active compound isolated from the Chinese herbal medicine *Salvia miltiorrhiza*. It is used to prevent and treat cardiovascular diseases. Yu et al. (2017) studied the effect and mechanism of Sal A at different doses on the BSCB permeability at different time points after SCI in rats. Studies have found that Sal A can promote the recovery of nerve function after SCI, which may be related to activating the miR-101/Cul3/Nrf2/HO-1 signaling pathway to repair BSCB.

#### Salidroside

Salidroside (Sal) is mainly found in the traditional Chinese medicine Rhodiola. Studies have shown that Sal has various pharmacological effects such as anti-fatigue, anti-aging, immune regulation, and scavenging free radicals (Wang C. et al., 2018; Su et al., 2019). It has been reported that Salidroside inhibits NF-κB, p38 and ERK signaling pathways, reduces inflammation damage in SCI rats, and promotes the recovery of motor function (Su et al., 2019). Gu et al. (2020) proved for the first time that Salidroside pretreatment can significantly improve the functional recovery of mouse spinal cord ischemia-reperfusion injury, and can significantly inhibit neuronal apoptosis, mainly by reducing apoptosis related to mitochondrial-dependent pathways. Among them, Salidroside’s antioxidant and autophagy-promoting properties play an important role (Gu et al., 2020). In addition, studies have also shown that Salidroside reduces oxidative stress in the spinal cord of SCI rats through the Nrf-2/HO-1 signaling pathway for promoting injury repair (Liu et al., 2021).

#### Asiatic Acid

Asiatic acid (AA) is a triterpenoid isolated from the plant *Centella asiatica*, and it has been widely used as an anti-inflammatory and antioxidant. Both *in vivo* and *in vitro* studies have shown that AA exerts a neuroprotective brain barrier that maintains blood stability and protects mitochondrial function (Mook-Jung et al., 1999; Lee et al., 2012). After exploring the therapeutic effect of AA on SCI (SCI) and its mechanism, Jiang et al. (2016) found that AA treatment resulted in an up-regulation of Nrf2/HO-1 levels in SC tissues and down-regulation of NLRP3 inflammasome protein expression. It is concluded that AA inhibits inflammation and oxidative stress by activating Nrf2 and HO-1 and inhibiting ROS and NLRP3 inflammasome pathways to achieve the purpose of inhibiting SCI (Jiang et al., 2016).

#### Curcumin

Curcumin (CUR) is the active ingredient of turmeric rhizomes and is the most abundant ingredient in turmeric. As we all know, CUR has a wide range of pharmacological activities, such as anti-inflammatory, anti-oxidant, anti-tumor, anti-apoptotic and other effects (Sirohi et al., 2017; Dai et al., 2018; Young-Seok et al., 2019). Evidence from emerging research shows that CUR can activate the Nrf2/HO-1 signaling pathway by increasing the activity of Nrf2, promote its antioxidant effect on free radicals, and down-regulate the activation of NF-κB by inhibiting P65 to exert its anti-inflammatory function (Jin W. et al., 2021).

#### Sulforaphane

The SF is an isothiocyanate extracted from broccoli and a natural inducer of the Keap1/Nrf2/ARE pathway, which can upregulate cytoprotective protein genes (Benedict et al., 2012). Previous experiments have found that the anti-inflammatory activity of SF is consistent with the increase in the expression of Nrf2-dependent genes (NQO1 and GST-a1) at 12 h after SCI (Mao et al., 2011), and is consistent with the increase in Nrf2 and GCL protein levels at 24 h after SCI (Wang et al., 2012). Benedict et al. (2012) pointed out that administration of sulforaphane (10 or 50 mg/kg) at 10 min and 72 h after contusive spinal cord injury significantly increased the expression of Nrf2/HO-1.

#### Rosmarinic Acid

Rosmarinic acid (RA) is a water-soluble polyphenol phytochemical, which is widely found in rosemary, sage, lemon balm and thyme. RA is a well-known natural antioxidant, with potential biological effects of scavenging free radicals and resisting oxidative stress and inflammation (Shang et al., 2017). Ma et al. (2020) used the rat SCI model and found that RA can significantly improve the recovery of exercise after SCI, reduce nerve defects, and reduce cell apoptosis. Therefore, it is concluded that the neuroprotective effect of RA on SCI may be related to its antioxidant and anti-inflammatory effects, which may be achieved by regulating the Nrf2/HO-1 and TLR4/NF-κb pathways. In addition, it was found that RA enhances its inhibitory effect on the NF-κB pathway by activating the Nrf2/HO-1 pathway.

#### Luteolin

Luteolin (LU) is a natural flavonoid with multiple targets. LU exists in a variety of plants, and these plants have higher contents in whole-leaf green orchid, pepper, wild chrysanthemum, honeysuckle, and perilla. Previous evidence has shown that LU has strong antioxidant, anti-inflammatory and other neuroprotective effects on SCII (Fu et al., 2021). Fu et al. (2018) explored whether LU can reduce SCII in rats. Experiments show that LU can resist oxidative stress, inhibit inflammatory damage and inhibit cell apoptosis by activating Nrf2 and inhibiting the NLRP3 inflammasome pathway. Then, by further demonstrating the neuroprotective effect of LU mediated by the Nrf2/GCL pathway in the SCCI model of transient abdominal aortic blockade in rats, it is confirmed that the neuroprotective effect of LU mainly depends on activating signal pathways such as Nrf2 (Fu et al., 2021).

#### Sinomenine

Sinomenine is an alkaloid that was originally isolated from the root of the plant sinomenine. In traditional Chinese medicine, it is used to treat rheumatoid arthritis (Gao et al., 2013). Zhang L. et al. (2019) found that an increase in Nrf2 translocation from cytoplasm to nucleus and Nrf2-mediated transactivation was observed after sinomenine administration, thereby achieving the purpose of inhibiting oxidative stress and inflammation. Experiments have proved that sinomenine can resist oxidative stress and anti-inflammatory damage after enhancing the Nrf2 signaling pathway, so it is expected to become a drug for the treatment of SCI (Zhang L. et al., 2019).

#### Ginsenoside Rb1

Ginsenoside Rb1 (G-Rb1) is a steroid compound, also known as triterpene saponins. Ginsenosides are regarded as the active ingredients in ginseng, and thus become the target of research. Evidence has shown that G-Rb1 has an antioxidant effect, scavenges free radicals, and improves immunity. Its mechanism has been used to treat various traumatic diseases (Chen et al., 2016; Dong et al., 2017). Previous experiments found that ginseng significantly improved SCI rats by regulating oxidative stress and inflammatory damage (Wang et al., 2015). Liu et al. (2018) pointed out that as an important active component of ginseng, G-Rb1 can reduce the oxidative stress that damages the spinal cord, and its mechanism may at least partly involve the eNOS/Nrf2/HO-1 pathway.

#### Gastrodin

Gastrodin (GAS) is known as Gastrodia in China. It is a traditional Chinese medicine with multiple pharmacological and neuroprotective effects. It has long been used to treat dizziness, epilepsy, and stroke, and has neuroprotective effects (Song et al., 2013). Studies have reported that Fang et al. (2016) found that gastrodin perfusion in the abdominal aorta can reduce spinal cord ischemia-reperfusion injury by promoting the antioxidant capacity of mitochondria and inhibiting inflammatory damage. Du et al. (2016) found that GAS may enhance the Nrf2-GCLc/GCLm signaling pathway, thereby improving oxidative stress and inflammatory damage, thereby reducing the permeability of the blood-spinal cord barrier in SCI rats and promoting the recovery of motor function in SCI rats.

#### Perillaldehyde

Perillaldehyde (PAH) is one of the effective components of traditional Chinese medicine plants. It is widely used and has many pharmacological activities such as anti-inflammatory, anti-oxidant, and protecting blood vessels and nerves. In addition, in different disease models, PAH exerts a protective effect by activating Nrf2 (Fuyuno et al., 2018), which proves that Nrf2 may be one of the potential targets of PAH. Zheng W. et al. (2021) established an SCII rat model and a BV2 microglia model induced by oxygen and glucose deprivation/reoxygenation, and found that PAH treatment upregulated the levels of Nrf2 and HO-1 in the spinal cord of SCII rats, confirming that PAH can activate Nrf2/HO -1 pathway, thereby inhibiting the activation of microglia and alleviating inflammation and oxidative stress.

#### Trehalose

Gong et al. (2022) experimentally confirmed that trehalose inhibits ferroptosis and ferroptosis-related inflammation by activating the Nrf2/HO-1 pathway, thereby reducing neuronal degeneration and iron accumulation. This provides new evidence supporting the neuroprotective role of inhibiting ferroptosis in SCI.

### Nuclear Factor Erythroid-2-Related Factor 2 and Western Medicine

In addition to some traditional Chinese medicines, many western medicines have also been studied and proposed by acting on the Nrf2 pathway to play the role of anti-oxidative stress and reduce inflammatory damage, so as to achieve the purpose of treating SCI. Table 2 shows the application of Nrf2 in Western Medicine.

**TABLE 2:** The therapeutic effect of western medicine on spinal cord injury.

Medicine	Model	Result	Mechanism	References
Liponin A4	Rats	p-Akt, Nrf2, and HO-1 expression↑	Activate the Akt/Nrf2/HO-1 signaling pathway	Lu T. et al., 2018
Hydrogen sulfide	Rats	Decreased release of cytokines TNF-α, IL-1β, IL-6, and HMGB1 in spinal cord microglia	Activate Nrf2/HO-1 signaling pathway	Chen et al., 2019
Hydrogen sulfide	Rats	NQO-1 and HO-1 expression↑	NaHS activates the Nrf2 signaling molecule, increases the nuclear translocation of Nrf2, and activates the transcription of downstream target genes	Xu et al., 2021
Aspirin	Rats	Nrf2, quinine oxidoreductase 1 and HO-1 expression↑, TNF-α, IL-6 expression↓	Activating the Nrf2/HO-1 signaling pathway inhibits the activation and apoptosis of astrocytes after SCI	Wei et al., 2018
Dexmedetomidine	Rats	IL-1, IL-6, and TNF↓, MDA activity↓ SOD activity↑, Nrf2, and HO-1↑	Activate Nrf2/HO-1 signaling pathway	Luo et al., 2020
Imatinib	Rats	IL-6, TNF-α and ROS↓, SOD↑	Activate the Nrf2/HO-1 signal pathway	Liu et al., 2020
Zinc	Rats	ROS and malondialdehyde↓, SOD activity↑, GSH-Px↑, ROS and malondialdehyde↓, SOD activity↑, GSH-Px↑, Nrf2 and Ho-1↑, nlrp3 expression↓	Activates the Nrf2/Ho-1 signal pathway	Li et al., 2020
Lithium	Rats	The expression of Nrf2 and HO-1↑	Activates the Nrf2/heme oxygenase-1 pathway to exert anti-inflammatory and antioxidant effects	Zhao et al., 2022
Tetramethylpyrazine	Rats	The expression of IL-1b, TNF-α, IL-18↓ reduces the permeability of the blood-spinal cord barrier	Activate Akt/Nrf2/HO-1 signaling pathway	Wang et al., 2016
Probucol	Rats	Nrf2, HO-1 and NQO1↑, inflammatory factors, IL-1β, IL-6, and TNF-α↓	Activate the Nrf2/ARE signal pathway	Yamashita and Matsuzawa, 2009
Riluzole	Rats	The expression of GFAP and NF-H↓	Activate the Nrf2/HO-1 signaling pathway	Zhou et al., 2017
Methane-rich saline	Rats	HO-1, SOD, catalase and GSH↑, glutathione disulfide, superoxide, hydrogen peroxide, malondialdehyde, 8-hydroxy-2-deoxyguanosine and 3-nitrotyrosine↓	Activate Nrf2 signaling pathway	Zhou et al., 2018
Metformin	Rats	Nrf2, HO-1, and NQO1↑	Activate the Nrf2/ARE signal pathway	Wu et al., 2021
Erythropoietin	Rats	Nrf2, NQO1↑, and glutathione transferase activity↑	Activate the Nrf2 signal pathway	Wang et al., 2020
Lipopolysaccharide	Rats	The expression of Nrf2, p-PI3K/PI3K and p-Akt/Akt↑, nuclear translocation of Nrf2↑	Activates the PI3K-Akt-Nrf2 signaling pathway	Longhi et al., 2011
2-(-2-benzofuranyl)-2-imidazoline	Rats	Nrf2 and HO-1↑	Activate the Nrf2/HO-1 signaling pathway	Li et al., 2013
Morin	Rats	Nrf2 and HO-1↑	Activate the Nrf2/HO-1 signaling pathway	Jin H. et al., 2021
Valproic acid	Rats	Nrf2 and Bcl-2 gene expression↓	Improve the motor function of the rat contusion model by changing the Mst1, Bcl-2, and Nrf2 gene expression	Lin et al., 2019
Maltol	Rats	Nrf2↑ Pink1/Parkin-mediated mitophagy in PC12 cells↑	Activate the Nrf2/Pink1/Parkin Pathway	Mao et al., 2022

#### Liponin A4

Liponin A4 (LXA4) is one of the main liposomes formed by mammalian cells. It has been proven to be an important anti-inflammatory mediator. It is called the “stop signal” of inflammatory damage and can promote the alleviation of inflammation. Lu T. et al. (2018) previously found that LXA4 protects SCI by activating the Akt/Nrf2/HO-1 signaling pathway.

#### Hydrogen Sulfide

Hydrogen sulfide (H_2_S) is an inorganic compound and has been considered a colorless, flammable, and water-soluble toxic and harmful gas. It plays an important regulatory role in many diseases, including inflammation, diabetes, hypertension, and neurodegenerative diseases (Wang, 2012). Chen et al. (2019) revealed that NaHS exerts anti-injury and anti-inflammatory effects by activating the Nrf2/HO-1 pathway. Wang (2010) clarified the physiological role of H_2_S in neurological diseases. Xu et al. (2021) found that H_2_S showed neuroprotective effects on SCI model rats, improved nerve injury symptoms, reduced inflammatory factor secretion, nerve cell apoptosis, etc., which were achieved by activating the Nrf2 pathway.

#### Aspirin

Aspirin is the most widely used antipyretic, analgesic and anti-inflammatory drug in the world. Aspirin has a variety of pharmacological effects by inducing the expression of HO-1 protein. Previously, there is evidence that aspirin has significant benefits for the clinical recovery of SCI rats (Kermani et al., 2016). Wei et al. (2018) employed a spinal cord contusion model in Sprague-Dawley rats and injected aspirin into the intraperitoneal cavity for 7 days. Experiments have found that aspirin inhibits the activation and apoptosis of astrocytes after SCI by activating the Nrf2/HO-1 signaling pathway, and exerts a neuroprotective effect.

#### Dexmedetomidine

Dexmedetomidine (DEX) is a potent α2-adrenergic receptor agonist. It is often used to calm patients during surgery. Luo et al. (2020) used DEX to intervene in the rat SCI model and observed the expression of different functional indicators and related proteins. It was found that DEXA can reduce the infiltration of inflammatory cells, resist oxidative stress damage, and affect nerve function by activating the Nrf2/HO-1 pathway, thereby having a protective effect on SCI (Luo et al., 2020).

#### Imatinib

Imatinib is a small molecule protein kinase inhibitor, often used in the treatment of chronic myelogenous leukemia and malignant gastrointestinal stromal tumors. A study found that imatinib can protect the blood–brain barrier and relieve inflammation after central nervous system injury (Hochhaus et al., 2016). Liu et al. (2020) pointed out that imatinib inhibits the oxidative stress response in SCI rats by activating the Nrf2/HO-1 signaling pathway, thereby inhibiting cell apoptosis and inflammatory damage.

#### Zinc

Many biochemical reactions and physiological changes in the body are related to zinc. Zinc is closely related to the growth and maturation of neurons and energy metabolism (Le et al., 2020). Relevant studies have shown that an appropriate amount of zinc has anti-oxidation, anti-apoptosis and immunomodulatory effects (Li et al., 2019). Previous experiments by Li et al. (2020) proved that zinc may inhibit inflammation and oxidative damage after SCI by activating the Nrf 2/Ho-1 pathway, indicating that it has a protective effect on SCI. Later, it was confirmed that zinc promotes the degradation of oxidative stress products and lipid peroxides through the Nrf2/HO-1 and GPX4 signaling pathways to inhibit ferroptosis in neurons (Ge et al., 2021).

#### Lithium

Lithium is a first-line drug for the treatment of bipolar disorder and provides neuroprotection in a variety of neurological diseases (Young, 2009; Huo et al., 2012). More and more evidences show that lithium has multiple effects, including neuroprotection, inflammation suppression, inducing the secretion of neurotrophic factors, and enhancing neurogenesis (Son et al., 2003; Chiu and Chuang, 2011; Zhang et al., 2018). Many experiments have pointed out that lithium has great potential in the treatment of acute SCI (Yick et al., 2004; Abdanipour et al., 2019). Wang F. et al. (2018) found that lithium has an inhibitory effect on neuronal apoptosis in adult rats after SCI. Zhao et al. (2022) pointed out that lithium exerts anti-inflammatory and antioxidant effects through the Nrf2/heme oxygenase-1 pathway, and promotes recovery after SCI.

#### Tetramethylpyrazine

Tetramethylpyrazine (TMP) is the most important biologically active ingredient extracted from *Ligusticum chuanxiong*. Due to its anti-inflammatory, antioxidant and neuroprotective activities, TMP has been effectively used to treat SCI (Li J. et al., 2021). Wang et al. (2016) studied the possible mechanism of the neuroprotective effect of ligustrazine on the rat model of SCI and found that TMP has neuroprotective effect on SCI by activating the Akt/Nrf2/HO-1 signaling pathway.

#### Probucol

Probucol is usually used clinically as a lipid-lowering drug to lower cholesterol and reduce atherosclerosis (Yamashita and Matsuzawa, 2009). Studies have found that probucol can inhibit neuronal apoptosis by inhibiting the mTOR signaling pathway after SCI (Zhou et al., 2016), indicating that probucol has the potential to be used in the treatment of SCI. Experiments by Zhou et al. (2017) found that after probucol treatment of injured rats, the levels of Nrf2, HO-1, and NQO1 increased significantly, while the levels of inflammatory factors, IL-1β, IL-6 and TNF-α decreased, which indicates that probucol exerts a neuroprotective effect after SCI by activating the Nrf2/ARE pathway.

#### Riluzole

Riluzole is a sodium channel blocker and glutamate inhibitor. It is often used to treat amyotrophic lateral sclerosis (ALS) neurodegeneration. It has been found to be safe and effective in people with SCI, which was proven in a phase 1 clinical trial (Nguyen et al., 2021). Daverey and Agrawal (2020) found that riluzole exerts a neuroprotective effect on astrocytes and white matter of SCI by activating the Nrf2/HO-1 signaling pathway.

#### Methane-Rich Saline

How oxidative stress causes neuroinflammation and chronic pain has been proven, and methane-rich saline (MS) may provide anti-inflammatory and antioxidant effects to reduce chronic inflammatory pain (Zhou et al., 2018). Previous experimental results have shown that 20 ml/kg of MS can significantly reduce the infarct size 72 h after SCI, inhibit oxidative stress, inflammatory damage, apoptosis, and inhibit microglia activation, and improve hindlimb nerve function (Wang W. et al., 2017). Wang L. et al. (2017) pointed out that MS repairs SCI by activating the antioxidant, anti-inflammatory and anti-apoptotic activities of the Nrf2 signaling pathway. Therefore, MS may become a new promising drug for the treatment of ischemic SCI.

#### Metformin

Metformin, an organic compound, is a first-line drug for the treatment of type 2 diabetes. At present, many experiments have proved that metformin plays a role in various central nervous system (CNS) diseases. Wu et al. (2021) machine team discovered that metformin has a new therapeutic effect on the recovery of SCI by regulating the activation of microglia and increasing their autophagy levels. Song et al. (2021) pointed out that metformin treatment from the subacute phase significantly improved the motor function of SCI mice. Wang et al. (2020) found that the role of metformin in nerve regeneration after SCI was probably related to stabilization of microtubules and inhibition of the excessive activation of Akt-mediated Nrf2/ARE pathway-regulated oxidative stress and mitochondrial dysfunction.

#### Erythropoietin

Erythropoietin (EPO) is a biologically active acid glycoprotein mainly produced by the kidneys. In addition to stimulating red blood cell precursors, it also has neurological effects. Studies have shown that adequate human erythropoietin (rhEPO) can inhibit the apoptosis of spinal cord anterior and posterior horn neurons after nerve root crush injury (Sekiguchi et al., 2003). Jin et al. (2014) studied the mechanism of EPO to protect neuron damage caused by traumatic SCI and found that rhEPO can significantly promote SCI by activating the Nrf2 signaling pathway.

#### Lipopolysaccharide

Lipopolysaccharide (LPS) is the main component of the cell wall of Gram-negative bacteria. Interestingly, low-dose LPS can induce a protective cross-tolerance state, which has a protective effect on subsequent injury (Jacob et al., 2016). Studies have shown that low-dose LPS pretreatment has a neuroprotective effect (Longhi et al., 2011; Li et al., 2013), and its mechanism has been proven to be through activation of the PI3K-Akt-Nrf2 signaling pathway, thereby reducing the rate of apoptosis and inhibiting Oxidative stress (Li W. et al., 2021).

#### Maltol

Mao et al. (2022) evaluated the effect of Maltol in the treatment of spinal cord injury. Experiments found that maltol stimulates the expression of Nrf2, promotes the re-translocation of Nrf2 from the cytoplasm to the nucleus, and mediates apoptosis in neuronal cell death after spinal cord injury. In addition, maltol treatment was found to enhance PINK1/parkin-mediated mitochondrial phagocytosis in PC12 cells, thereby aiding the recovery of mitochondrial function.

In addition to the above-mentioned drugs, there are also drugs such as 2-(-2-benzofuranyl)-2-imidazoline (Lin et al., 2019), Morin (Jin H. et al., 2021), Valproic Acid (Mardi et al., 2021), etc. After research, it has been found that they can act by activating the Nrf2 signaling pathway. It has anti-inflammatory, oxidative stress and neuroprotective effects to achieve the purpose of treating SCI, and we will not summarize them one by one here.

### Non-drug Treatment

#### Stem Cell Transplantation

Studies have shown that bone marrow mesenchymal stem cell (BMSC) transplantation is a promising strategy for spinal cord injury (SCI) repair. Polydatin is a key component of the traditional Chinese medicine *Polygonum cuspidatum*. It has a significant neuroprotective effect on various central nervous system disorders and can protect bone marrow mesenchymal stem cells from oxidative damage. Zhan et al. (2020) found that PD enhances the neuronal differentiation of bone marrow mesenchymal stem cells and promotes functional recovery through Nrf2 activation, indicating that this is a promising new method for the treatment of spinal cord injury. In addition, Yang et al. (2016) found that BMSC combined with plumbagin can alleviate SCI through its anti-oxidative stress, inflammation, apoptosis and activation of the Nrf2 pathway.

#### Diet Therapy

The ketogenic diet (KD) is high in fat, while the carbohydrate content is kept to a minimum. There is recent convincing evidence that KD has neuroprotective effects in animal models of neurological diseases, which has aroused interest in testing the benefits and mechanism of action of this diet (Gough et al., 2021). Studies by Seira et al. (2021) have shown that KD can save mitochondrial function and improve metabolism after SCI, suggesting that KD has the potential to treat acute SCI. The ketogenic diet such as Lu Y. et al. (2018) alleviates the oxidative stress and inflammatory damage after spinal cord injury by activating Nrf2 and inhibiting the NF-κB signaling pathway.

#### Physiotherapy

Electroacupuncture (EA) is widely used in various acute and chronic diseases, and has been proven to have a good effect on central nervous system diseases, especially spinal cord injury (Liu et al., 2010; Yan et al., 2011). Early studies by Dai et al. (2019) found that electroacupuncture can significantly improve the inflammatory response and oxidative stress response in SCI mice, inhibit the excessive proliferation of astrocytes, and promote the repair of spinal cord function. After studying its mechanism in the later stage, it is concluded that electroacupuncture can inhibit inflammation and oxidative stress in mice with spinal cord injury by activating ApoE and Nrf2 (Dai et al., 2021).

#### Hyperbaric Oxygen

Hyperbaric oxygen (HBO) is a physical method of treating hypoxic diseases with high-concentration oxygen. Studies have shown that hyperbaric oxygen therapy can reduce spinal cord injury and improve nerve function (Sun et al., 2019; Ying et al., 2019). The oxidative stress induced epithelial cell damage caused by spinal cord injury leads to epithelial barrier dysfunction. Therefore, Liu et al. (2021b) studied this and found that hyperbaric oxygen therapy promotes the Nrf2 signaling pathway to produce antioxidant effects on intestinal epithelial barrier dysfunction after spinal cord injury.

## Conclusion and Outlook

This article briefly summarizes the regulatory mechanism and treatment methods of the Nrf2 pathway in SCI. SCI is a devastating nervous system damage. With the expansion of human activities, the incidence of SCI is gradually increasing, so it is very important to explore effective treatment measures. The clinical and experimental studies discussed in this article have proved that the main cause of neuronal damage and death in SCI is the occurrence of secondary damages such as oxidative stress and inflammation. Therefore, suppression of secondary damage is the focus of treatment.

As a pleiotropic transcription factor, Nrf2 can induce a defense mechanism against oxidative stress and inflammatory damage, and regulate the expression of related genes. The regulated coded HO-1 achieves anti-inflammatory goals through enzymatic degradation of pro-inflammatory free heme and increasing the levels of bilirubin and carbon monoxide. In addition, Nrf2 itself can negatively regulate inflammatory mediators, such as chemokines, cytokines, COX-2, iNOS, etc., and reduce the activity of NF-κB to achieve anti-inflammatory effects. Nrf2 also regulates genes related to antioxidant mechanisms, such as the synthesis of GSH, elimination of ROS, drug delivery, and detoxification of exogenous drugs, thereby inducing the expression of many cytoprotective genes during oxidative and electrophilic stress responses. Through the regulation of Nrf2 pathway, it plays the role of anti-oxidative stress and inflammatory damage to effectively treat SCI, which is a direction of future research. However, the effectiveness of drugs and non-drug therapies involving the Nrf2 pathway in the treatment of spinal cord injury remains to be verified. On this basis, the Nrf2 signaling pathway should be further studied, with a view to discovering more highly targeted drugs and non-drug therapies to combat oxidative stress and inflammatory damage, thereby effectively inhibiting and treating spinal cord injury and other neurological dysfunctions.

The mechanism and treatment of spinal cord injury have been explored for a long time; however, the effective repair of damaged neural function by anti-oxidative stress and the occurrence of inflammatory responses remains a great challenge. At present, the treatment methods of SCI are mainly through early surgical intervention and drug treatment, as well as some unconventional treatment methods, such as electroacupuncture, hyperbaric oxygen, stem cell therapy, etc., but the effects are uneven. On the basis of understanding the pathological changes of SCI, it has become a difficult task to conduct a deeper and broader exploration of efficient treatment measures. With the development of science and technology and the continuous exploration of human beings, we believe that SCI research will soon have further breakthroughs.

## Author Contributions

TJ designed the theme of the manuscript, wrote all the chapters, and created all the tables and graphs. YH made critical revisions to the manuscript. Both authors contributed to the article and approved the submitted version.

## Conflict of Interest

The authors declare that the research was conducted in the absence of any commercial or financial relationships that could be construed as a potential conflict of interest.

## Publisher’s Note

All claims expressed in this article are solely those of the authors and do not necessarily represent those of their affiliated organizations, or those of the publisher, the editors and the reviewers. Any product that may be evaluated in this article, or claim that may be made by its manufacturer, is not guaranteed or endorsed by the publisher.
